# Converging Pharmacological and Genetic Evidence Indicates a Role for Steroid Sulfatase in Attention

**DOI:** 10.1016/j.biopsych.2009.01.001

**Published:** 2009-08-15

**Authors:** William Davies, Trevor Humby, Wendy Kong, Tamara Otter, Paul S. Burgoyne, Lawrence S. Wilkinson

**Affiliations:** a Behavioural Genetics Group, School of Psychology and Department of Psychological Medicine, School of Medicine, University of Cardiff, London, United Kingdom; b Department of Anatomy, University of Bristol, London, United Kingdom; c Division of Stem Cell Biology and Developmental Genetics, Medical Research Council National Institute for Medical Research, London, United Kingdom

**Keywords:** Accuracy, attention-deficit/hyperactivity disorder, dehydroepiandrosterone sulfate, 5-choice serial reaction time task, Turner syndrome, X chromosome

## Abstract

**Background:**

Attention-deficit/hyperactivity disorder (ADHD) is a complex neurodevelopmental disorder characterized by deficits in attention, increased motor impulsivity, and hyperactivity. Preliminary work in mice and humans has suggested the X-linked gene *STS* (which encodes the enzyme steroid sulfatase) as a mediator of attentional functioning and as a candidate gene for ADHD.

**Methods:**

The effects of modulating the murine steroid sulfatase axis pharmacologically (through administration of the substrate dehydroepiandrosterone sulfate [DHEAS], 0–40mg/kg, or acute inhibition of the enzyme by COUMATE, 10mg/kg) or genetically (through loss of the gene in 39,X^Y^*O mice) were assayed using the 5-choice serial reaction time task (5-CSRTT) a test of visuospatial attention and response control, and a locomotor activity paradigm.

**Results:**

DHEAS administration improved 5-CSRTT performance under attentionally demanding conditions, whereas steroid sulfatase inhibition impaired accuracy under the same conditions. Loss of *Sts* expression constitutively throughout development in 39,X^Y^*O mice resulted in deficits in 5-CSRTT performance at short stimulus durations and reduced anticipatory responding. Neither the pharmacologic nor the genetic manipulations affected basic locomotor activity.

**Conclusions:**

These data provide converging evidence indicating a role for steroid sulfatase in discrete aspects of attentional functioning and are suggestive of a role in motor impulsivity. The findings provide novel insights into the neurobiology of attention and strengthen the notion of *STS* as a candidate gene for the attentional component of ADHD.

Attention-deficit/hyperactivity disorder (ADHD) is a neurodevelopmental disorder characterized by deficits in attention, hyperactivity, and increased motor impulsivity (1). Although environmental factors may play a role in precipitating the disorder (2), there is also a strong underlying genetic component (3). Diagnosis of ADHD is approximately 4 times more common in males than in females (4), and the etiology of the disorder is sex-specific (5–7); therefore, genes on the sex chromosomes may influence susceptibility.

Evidence for an X-linked gene influencing attention has come from work in Turner syndrome (TS) subjects. Individuals with TS are females who, most typically, lack a whole X chromosome. They are impaired on neuropsychological and real-life tests of attention (8–10) and are at increased ADHD risk relative to 46,XX subjects (11). We have previously shown that female mice with a single X chromosome (39,XO) also show attentional deficits relative to their 40,XX litter mates (12). Additionally, we showed that 40,XY*^X^ mice (essentially 39,XO mice bearing a small number of extra genes on the Y*^X^ chromosome) do not differ from 40,XX mice in terms of their attentional performance, implying that Y*^X^ genes can influence attention. We argued that *Sts*, encoding the enzyme steroid sulfatase, represents the best candidate for this “rescue” effect on the basis of its expression pattern and function (12). In humans, as in mouse, *STS* escapes X-inactivation (13); thus, it may be regarded as a candidate gene for the TS attentional deficits.

Steroid sulfatase converts various sulfated compounds to their nonsulfated forms, notably the neurosteroid dehydroepiandrosterone sulfate (DHEAS) to DHEA (14); DHEAS and DHEA have important effects on neural function, including cognition (15). In the mouse brain, *Sts* is most highly expressed in the cortex, hindbrain, and thalamus, with lower expression elsewhere (16). Levels peak around the perinatal period and are relatively low thereafter (17). In humans, *STS* is expressed in the neocortex (18). Steroid sulfatase influences the malignancy of hormone-dependent cancers (probably by enhancing the availability of free steroid precursors). Consequently, substantial effort has been invested in developing specific inhibitors of the enzyme for therapy, for example, the arylsulfamate-based compound COUMATE (19,20). In mice, systemic COUMATE administration results in profound attenuation (∼ 70%) of brain steroid sulfatase activity within 24 hours (21).

Previous data have implicated *STS* in ADHD risk: males with cytogenetic deletions encompassing the gene (or with inactivating mutations within it) appear to show an enhanced vulnerability to the disorder (22–25). However, the pleiotropic effects of multiple gene deletions, the small sample sizes used, and the lack of appropriate control samples means that the conclusions of these studies should be interpreted cautiously. We directly tested the hypothesis that steroid sulfatase could influence ADHD endophenotypes (attention, motor impulsivity, and activity) using mouse models. Two approaches were employed: in a pharmacologic approach, adult mice were given DHEAS or COUMATE to ascertain the effects of acute manipulation of the steroid sulfatase axis. Because ADHD can persist into adulthood with adverse consequences (26), such an approach could shed light on the molecular pathogenesis of adult ADHD. In a parallel genetic approach, 39,X^Y^*O male mice (27) were compared with 40,XY males. 39,X^Y^*O mice have a single large sex chromosome comprising the X and Y attached via an end-to-end fusion of their pseudoautosomal regions, with both copies of the *Sts* gene deleted but a normal complement of all other X and Y genes (28) [Figure 1). Using this model, the effects of loss of steroid sulfatase function throughout development could be determined.

Attention was assayed using the 5-choice serial reaction time task (5-CSRTT) (29) in which subjects must respond as accurately and as rapidly as possible with a directed nose-poke response to the presentation of a light stimulus presented pseudo-randomly in one of five spatial locations to gain a reinforcer. This task also provides an index of motor impulsivity in that it is possible to measure the ability of the subjects to withhold responding in a pause (the “intertrial interval”) before the onset of the stimulus. The neural substrates underlying 5-CSRTT performance have been well specified in rodents (30).

On the basis of our previous findings (12), the main prediction at the outset of the experiments was that pharmacologic or genetic manipulations (or both) influencing the steroid sulfatase axis would result in alterations in attention.

## Methods and Materials

### Subjects

For the pharmacologic study, we used male MF1 mice (Harlan, Bicester, United Kingdom). Behavioral testing commenced at approximately 5 months of age. For the genetic study, 40,XY and 39,X^Y^*O male mice on identical genetic backgrounds were imported from Medical Research Council National Institute for Medical Research, United Kingdom. 39,X^Y^*O mice were produced from two separate crosses: 39,X^*Paf*^O x 40,XY* and 39,X^*Paf*^O x 39,X^Y^*O (see Supplement 1). Behavioral testing commenced at 4–6 months of age. Mice were treated in accordance with the Animal Scientific Procedures Act (United Kingdom, 1986). For details of animal husbandry, see Supplement 1.

### Reinforcer Preference, 5-CSRTT Training to Stable Baseline Performance

Reinforcer preference was carried out as described previously (29), with the main index of preference being the amount of reinforcer (10% condensed milk solution, Nestle, Croydon, United Kingdom) drunk on the final day of preference testing as a percentage of total fluid consumption (choice between water and reinforcer). The 5-CSRTT apparatus, behavioral shaping, trial design, and training are described in detail elsewhere (12) (see also Supplement 1). At baseline stimulus durations of 1 sec, mice performed more than 50 trials at greater than 80% accuracy (i.e., correct:incorrect responses) and less than 30% omissions (i.e., no response to stimulus presentation); the intertrial interval (ITI) was set to 5 sec.

### 5-CSRTT Manipulations

At stable baseline performance (baseline criteria for ≥ 5 consecutive days), three behavioral manipulations designed to affect different aspects of attentional load/impulse control were performed with 2 or more days of consecutive baseline performance between each manipulation session: 1) short stimulus durations (.25, .5, .75 and 1.0 sec), 2) extra-short stimulus durations (.1, .3, .5, and .7 sec; short stimulus durations increasing attentional load), and 3) long ITI (5, 6, 7, and 8 sec; long ITIs taxing the ability to withhold responding). During manipulation sessions, the various parameters were presented pseudo-randomly. Where relevant, following completion of these manipulation sessions in the 5-CSRTT, the extent to which any effects on behavior could be attributed to attentional processes was further assessed in a 1-choice version of the task (1-CSRTT) in which attentional demands of the task were reduced by only presenting the stimulus in the central response hole (12,29). Stimulus duration manipulations were performed after five or more sessions at criterion on the modified task.

### Behavioural Measures on the 5-CSRTT/1-CSRTT

The following behavioral measures were recorded: number of trials, accuracy and omissions, (indexing failures of detection and/or motivational/motor deficits) (30), number of premature responses (i.e., nose pokes made in the ITI before stimulus presentation, indexing motor impulsivity) (30), correct response latency (defined as the time taken to nose poke in the illuminated hole following stimulus onset), and the latency to collect and consume the reinforcer.

### Pharmacological Analysis in the 5-CSRTT/1-CSRTT

In the first part of the pharmacologic study, DHEAS (Sigma-Aldrich, Gillingham, United Kingdom) was administered to mice (*n* = 12) at stable baseline performance on the 5-CSRTT 1 hour before behavioral testing. Each mouse received four doses of drug (0, 5, 15, and 40 mg/kg in distilled water intraperitoneal [i.p.]) with at least 72 hours washout between treatments; the order in which the doses were given was pseudo-randomized to negate possible order effects. The drug doses selected have previously been shown to influence behavior in mice (21,31). Subsequently, each mouse was given either vehicle or 40 mg/kg DHEAS (pseudo-randomized) and tested specifically on the 5-CSRTT short-stimulus-duration manipulation; following washout, mice were given the opposite drug/vehicle treatment and retested on the task manipulation. In the second part of the study, mice (*n* = 12) were pseudo-randomly injected either with vehicle (.5% methylcellulose, .9% NaCl in distilled water per os [p.o.]) or with COUMATE (10 mg/kg in the same vehicle p.o., COUMATE made according to [21]) 24 hours before behavioral testing on the short-stimulus-duration manipulation in the 5-CSRTT. After at least 72 hours washout, mice were given the opposite drug/vehicle treatment and retested. The effects of COUMATE administration were then tested in the 1-CSRTT under the same conditions.

### Locomotor Activity

Locomotor activity was measured in dim lighting (5 lux) using cages each fitted with two infrared beams. Mice were allowed to explore freely for a 1-hour session, with the number of individual infrared beam breaks indexing their activity. For the pharmacologic study, mice (*n* = 7) were run for 6 days (1 session per day) to habituate them to the apparatus and to limit any interactions between drug treatment and reactivity to novelty. They were then treated with either vehicle or COUMATE 24 hours before testing. After washout, the mice were given the opposite treatment and rerun. The 40,XY (*n* = 20) and 39,X^Y^*O (*n* = 14) mice were run on 3 consecutive days to ascertain the effects of *Sts* loss on reactivity to a novel environment and basic locomotor activity.

### Statistical Analysis

Statistical analysis was performed using SPSS12. Data were subject to *t* test or repeated-measures analysis of variance (ANOVA) if normal, with factors Drug Treatment/Genotype and Task Manipulation/Day or Mann-Whitney *U* test and Wilcoxon Signed-Ranks test (if not normal). *t* tests, Mann-Whitney *U* tests, and Wilcoxon Signed-Ranks tests were used to analyze overall performance in the task. Percentage data (accuracy and omissions) and premature response data were arcsine or square root transformed, respectively, before ANOVA (32). If ANOVA indicated a significant interaction between factors, post hoc pairwise comparisons were performed using Tukey's honestly significant difference test. Values of *p* < .05 were regarded as significant. The results were not corrected for multiple testing. Data are presented as mean values ± standard error of the mean.

## Results

### Pharmacologic Study

#### DHEAS Does Not Influence Baseline Performance in the 5-CSRTT

DHEAS administration at any of the doses tested did not significantly affect the main 5-CSRTT performance measures at baseline (Supplement 1).

#### DHEAS Reduces Omissions Under Attentionally Demanding Conditions

**Figure 2:**
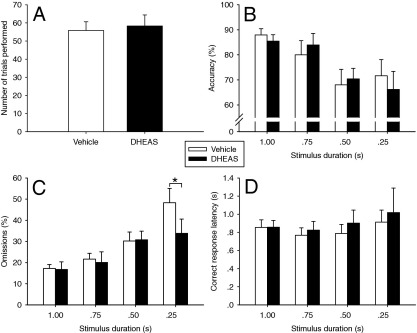
Effects of dehydroepiandrosterone sulfate (DHEAS) administration on behavioral indices of attention during a 5-choice serial reaction time task manipulation in which stimulus durations were reduced. The drug treatment did not significantly affect the number of trials performed **(A)** or accuracy at any value of stimulus duration **(B)**. However, DHEAS did result in fewer omissions at the shortest, and therefore, most attentionally demanding, time point (*p < .05) **(C)**. There were no significant effects of the treatment on the latency taken to make at correct choice at any value of stimulus duration (**D**).

We tested whether the highest dose of DHEAS (40 mg/kg) would influence behavior under more attentionally demanding conditions (i.e., attenuated stimulus durations). As at baseline, DHEAS administration did not affect trial number [Drug Treatment, *t*(11) = −.58, ns, Figure 2A], nor did it influence accuracy [Drug Treatment, *F*(1,11) = .03, ns, Drug Treatment × Task Manipulation, *F*(3,33) = .82, ns, Figure 2B]. However, DHEAS treatment did appear to enhance task performance specifically at the shortest stimulus duration of .25 sec, as reflected in the significant Drug Treatment × Task Manipulation interaction on omissions [*F*(3,33) = 3.23, *p* = .035] and subsequent post hoc test (*p* < .05; Figure 2C). DHEAS did not affect correct response latency under the shorter stimulus conditions [Drug Treatment, *F*(1,11) = .76, ns, Drug Treatment × Task Manipulation, *F*(3,33) = .25, ns, Figure 2D], nor the total number of premature responses made [Drug Treatment, *t*(11) = −1.34, ns].

#### COUMATE Impairs Accuracy in the 5-CSRTT Under Attentionally Demanding Conditions; This Impairment Is Alleviated in the 1-CSRTT

**Figure 3:**
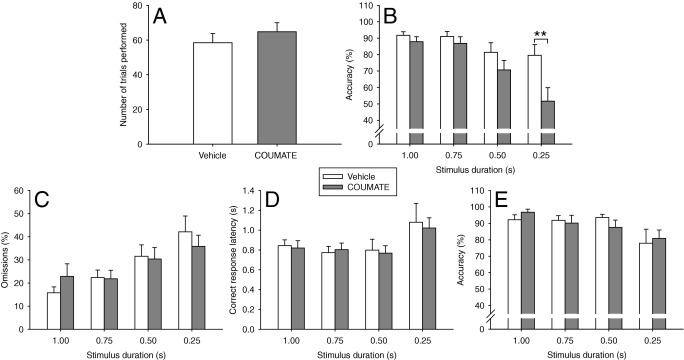
Effects of steroid sulfatase inhibition by COUMATE administration on behavioral indices of attention during 5-choice (5-CSRTT) and 1-choice serial reaction time task (1-CSRTT) manipulations in which stimulus durations were reduced. The drug treatment did not affect total number of trials performed in the 5-CSRTT **(A)** but did have significant effects on accuracy **(B)**; this drug effect was present at all stimulus durations but was most marked at the most attentionally demanding stimulus duration of .25 sec (**p < .01). The drug manipulation had no effects on omissions **(C)** or correct response latencies **(D)**. Importantly, when the attentional demands of the task were reduced in the 1-CSRTT, COUMATE administration had no effect on accuracy **(E)**.

COUMATE administration resulted in significantly decreased accuracy under short-stimulus-duration conditions [Drug Treatment, *F*(1,11) = 8.87, *p* = .013]; this deficit in accuracy was particularly pronounced at the shortest .25-sec stimulus duration [Drug Treatment × Task Manipulation, *F*(3,33) = 3.79, *p* = .019, subsequent pairwise comparison *p* < .01; Figure 3B]. The effects of the drug manipulation were behaviorally specific in that there were no effects of Drug Treatment on trial number [*t*(11) = −1.34, ns, Figure 3A), omissions [*F*(1,11) = .01, ns, Drug Treatment × Task Manipulation, *F*(3,33) = 1.47, ns, Figure 3C], correct response latency [*F*(1,11) = .14, ns, Drug Treatment × Task Manipulation, *F*(3,33) = .11, ns, Figure 3D] or total number of premature responses (Vehicle vs. COUMATE, 3.2 ± 1.0 vs. 5.2 ± 1.6, W = −27, Z = −1.33, ns). Furthermore, Drug Treatment did not influence two measures of motivation: the latency to collect and consume the reinforcer [*F*(1,11) = .18, ns, and *F*(1,11) = .25, ns, respectively].

Baseline performance on the 1-CSRTT was, as expected, superior to that on the 5-CSRTT (accuracy: > 95%, omissions: < 15%, correct response latency: ∼.85 sec). In the 1-CSRTT, COUMATE did not affect accuracy [Drug Treatment, *F*(1,11) = .02, ns; Drug Treatment × Task Manipulation, *F*(3,33) = .89, ns; Figure 3E]. There were no effects of Drug Treatment on omissions [*F*(1,11) = .00, ns, and Drug Treatment × Task Manipulation, *F*(3,33) = .70, ns] or correct response latency [*F*(1,11) = .89, ns, Drug Treatment × Task Manipulation, *F*(3,33) = .56, ns].

### Genetic Study

#### 40,XY and 39,X^Y^*O Mice Demonstrate Equivalent Reinforcer Preference, 5-CSRTT Acquisition, and Baseline Performance

The 40,XY (*n* = 9) and 39,X^Y^*O (*n* = 12) mice were trained to performance criteria in the 5-CSRTT. The groups displayed equivalent reinforcer preference [78.8 ± 5.5% vs. 84.7 ± 4.8%, respectively, GENOTYPE, *t*(19) = −.81, ns] and acquired the task in an equal number of sessions, indicating no effects of Genotype on learning [68.4 ± 8.1 vs. 63.6 ± 8.8, respectively, *t*(19) = .39, ns]. At baseline, 40,XY and 39,X^Y^*O mice generally performed equivalently (Table 1), although the 39,X^Y^*O mice adopted a more efficient response strategy, performing fewer nose pokes per trial than 40,XY subjects.

#### 39,X^Y^*O Mice Show Increased Omissions Relative to 40,XY Mice Under Attentionally Demanding Conditions; This Group Difference Is Abolished in the 1-CSRTT

**Figure 4:**
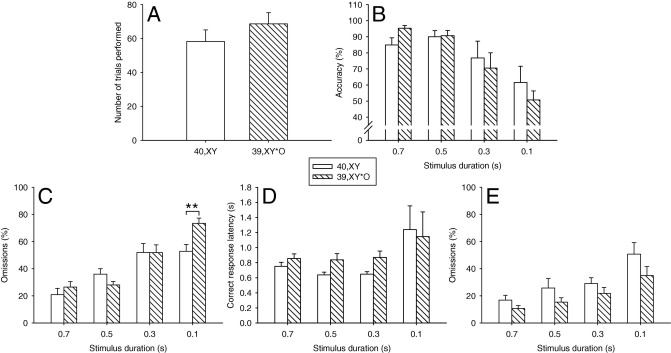
Effects of *Sts* gene loss in 39,X^Y^*O mice on behavioral indices of attention during 5-choice (5-CSRTT) and 1-choice serial reaction time task (1-CSRTT) manipulations in which stimulus durations were reduced. The two groups performed equal numbers of trials in the 5-CSRTT **(A)** and to the same degree of accuracy **(B)**. At the shortest stimulus duration, 39,X^Y^*O mice omitted more trials than 40,XY mice (**p < .01) **(C)**. No effects of the gene manipulation were observed on correct response latencies for any value of stimulus duration **(D)**. When the attentional demands of the task were reduced in the 1-CSRTT, 39,X^Y^*O mice performed equivalently to 40,XY mice in terms of omissions at the shortest stimulus duration **(E)**.

To further tax attention, we compared the performance of 40,XY and 39,X^Y^*O mice on two manipulations in which stimulus durations were attenuated: one with stimulus durations ranging from 1.0 to .25 sec and a second, more attentionally demanding manipulation, with stimulus durations ranging from .7 to .1 s. On the first manipulation, there was no effect of Genotype with respect to the two main indices of attention, accuracy and omissions [*F*(1,19) = .31, ns, and *F*(1,19) = .00, ns, respectively], although there was a trend for 39,X^Y^*O mice to make more omissions than 40,XY mice at the shortest stimulus duration (Genotype × Task Manipulation, *F*(3,57) = 1.54, ns]. On the second manipulation, this effect on omissions was more pronounced, giving rise to a significant Genotype × Task Manipulation interaction *F*(3,57) = 4.53, *p* < .01] and a significant pairwise comparison at the shortest stimulus duration of .1 sec (*p* < .01; Figure 4C). Importantly, this effect occurred in the absence of any Genotype effects on markers of motivation including trial number [*t*(19) = −1.07, ns, Figure 4A], correct response latency [*F*(1,19) = .43, ns, Genotype × Task Manipulation, *F*(3,57) = .45, ns, Figure 4D], latency to collect the reinforcer [*F*(1,19) = 1.39, ns, Genotype × Task Manipulation, *F*(3,57) = 1.38, ns], and latency to consume the reinforcer [*F*(1,19) = 1.46, ns, Genotype × Task Manipulation, *F*(3,57) = 2.02, ns]. Although accuracy did not differ significantly between genotypes on this latter manipulation [Genotype, *F*(1,19) = .02, ns], there was a trend toward impaired performance in the 39,X^Y^*O group at the shortest stimulus duration of .1 s [Genotype × Task Manipulation, *F*(3,57) = 1.71, ns, Figure 4B].

**Figure 5:**
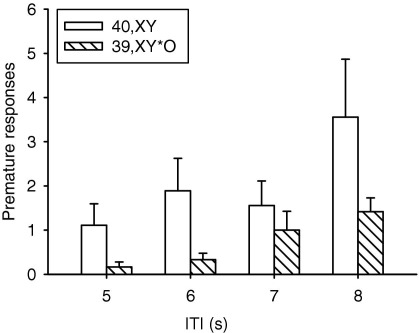
Effects of *Sts* gene loss in 39,X^Y^*O mice on an index of motor impulsivity (premature responses) on a 5-choice serial reaction time task (5-CSRTT) manipulation in which the intertrial interval (ITI) between trial initiation and stimulus presentation was extended. 39,X^Y^*O mice made fewer premature responses than 40,XY mice for all values of ITI.

Baseline performance of 40,XY and 39,X^Y^*O mice in the 1-CSRTT was equivalent and superior to that in the 5-CSRTT (Table 2). In the 1-CSRTT, there were no stimulus duration-dependent group effects on omissions [Genotype, *F*(1,19) = 3.15, ns; Genotype × Task Manipulation, *F*(3,57) = .97, ns, Figure 4E]. Furthermore, accuracy did not differ significantly between the groups on the 1-CSRTT short-stimulus-duration manipulation [Genotype, *F*(1,19) = 1.18, ns; Genotype × Task Manipulation, *F*(3,57) = .13, ns]. However, 39,X^Y^*O mice tended to respond correctly more slowly for all values of stimulus duration than 40,XY mice [Genotype, *F*(1,19) = 5.66, *p* = .03, Genotype × Task Manipulation, *F*(3,57) = 1.47, ns]. The only other significant effect of Genotype was in the “long ITI” 5-CSRTT manipulation. As expected, premature responding increased with increased ITI length [Task Manipulation, *F*(3,57) = 6.92, *p* < .0005], but unexpectedly 39,X^Y^*O mice made fewer anticipatory responses than 40,XY mice for all values of ITI [Genotype, *F*(1,19) = 4.39, *p* = .05, Genotype × Task Manipulation, *F*(3,57) = .32, ns, Figure 5] suggesting that the former group display a reduced tendency for impulsive responding.

#### Steroid Sulfatase Manipulations Do Not Affect Locomotor Activity

**Figure 6:**
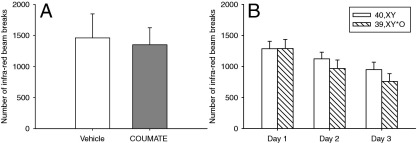
Effects of acute inhibition of steroid sulfatase by COUMATE, or loss of the *Sts* gene in 39,X^Y^*O mice, on locomotor activity. COUMATE administration had no effect on locomotor activity **(A)**. The 39,X^Y^*O mice showed equivalent levels of activity to 40,XY mice on being placed in a novel environment, and both groups habituated to the environment equally over 3 consecutive days of testing **(B)**.

COUMATE treatment did not have any effect on locomotor activity [Drug Treatment, *t*(6) = .8, ns, Figure 6A]. Initial activity levels of 40,XY and 39,X^Y^*O mice were identical and decreased equally across sessions [Genotype: *F*(1,32) = .45, ns; Genotype × DAY: *F*(2,64) = 1.85, ns, Figure 6B].

## Discussion

Previous studies have suggested that the enzyme steroid sulfatase, encoded by the X-linked gene *STS*, and a modulator of neuroactive steroid activity (15), may play a role in attention (12,23). We tested this idea using pharmacologic and genetic approaches in mouse models.

We hypothesized that haploinsufficiency for steroid sulfatase could account for attentional deficits in 39,XO mice through limiting the conversion of DHEAS to DHEA (12). Hence, we expected that DHEAS administration (which leads to a rise in brain DHEAS and DHEA within 1 hour) (33) may act to improve aspects of attention. DHEAS administration did indeed improve 5-CSRTT performance at the shortest stimulus duration. Whether it is DHEAS or one of its downstream metabolites (e.g., DHEA or androstenediol) that causes this effect remains to be clarified, but this finding is consistent with work showing that DHEA augmentation in schizophrenic patients improves sustained attention (34). Because methylphenidate treatment has been shown to increase systemic DHEA levels in ADHD subjects (35), we propose that DHEA administration may also improve attentional function in ADHD.

We further anticipated that inhibition of steroid sulfatase would elicit deficits on the 5-CSRTT that recapitulated those seen in the 39,XO mouse. Like 39,XO mice, COUMATE-treated mice showed specific impairments in accuracy at short stimulus durations on the 5-CSRTT, and, as in 39,XO mice, these drug-induced deficits were rescued in the less attentionally demanding 1-CSRTT. Together these findings suggest that steroid sulfatase influences ongoing attentional processes. Moreover, the data indicate that steroid sulfatase plays a similar role in attention in both male (COUMATE-treated) and female (39,XO) mice.

The 39,X^Y^*O mice, which lack steroid sulfatase expression throughout development, demonstrated impaired 5-CSRTT performance (significantly increased omissions and a nonsignificant trend toward impaired accuracy at short stimulus durations) relative to 40,XY control mice; these deficits were rescued in the 1-CSRTT. As 39,X^Y^*O mice performed equivalently to 40,XY mice at longer stimulus durations, and because the two groups of mice were equally motivated, these results may again reasonably be interpreted as a specific impairment in attention. These data, together with previous findings in other models, support the conclusion that alterations in steroid sulfatase function can lead to specific changes in performance on the 5-CSRTT and that these changes are likely to reflect attentional effects. Future work may assess the effects of DHEAS and COUMATE administration in 39,X^Y^*O mice on 5-CSRTT performance to determine the extent to which the behavioral effects mediated by these compounds are truly Sts-dependent.

We note the discrepancy between the measures influenced by acute inhibition of steroid sulfatase in COUMATE-treated mice and constitutive loss of the *Sts* gene in 39,X^Y^*O mice (effects on accuracy and omissions, respectively). Such a discrepancy may arise because of enzyme inhibition via COUMATE causing acute, and only partial, loss of function, whereas *Sts* deletion causes complete loss of the enzyme throughout development. Interestingly, a similar dissociation between the effects of acute pharmacologic and genetic manipulations of the cholinergic axis on accuracy and omissions has been described previously (36). Although both accuracy and omissions may be regarded as indices of attention, the precise relationship between the two measures, and the extent to which they index common psychology and neurobiology in rodents, is unclear.

We also tested whether steroid sulfatase influences other ADHD endophenotypes. Pharmacologic manipulations of the steroid sulfatase axis had no effect on motor impulsivity (as indexed by the degree of anticipatory responding), whereas constitutive loss of *Sts* in 39,X^Y^*O mice resulted in both significantly fewer nose pokes per trial and reduced premature responding. Because ADHD is characterized by increased motor impulsivity and because the incidence of hyperactive-impulsive type ADHD in Turner syndrome is elevated (11), these latter results are surprising. The dissociation between acute drug effects and *Sts* loss may reflect the differential effects of steroid sulfatase on ongoing and developmental processes, respectively. In terms of developmental effects, DHEA is known to influence neurogenesis (37). The dissociable effects of steroid sulfatase manipulations on attention and impulse control suggest that it would be interesting to perform other assays of response inhibition (e.g., stop-signal or delayed reinforcement paradigms) (38, 39). Neither steroid sulfatase inhibition nor *Sts* loss resulted in effects on locomotor activity. These data, in conjunction with previous data showing an absence of effects of 40mg/kg DHEAS on locomotor activity in mice (40), indicate that steroid sulfatase is unlikely to influence neurobiological pathways underpinning activity. ADHD is often comorbid with conduct disorder and oppositional-defiant disorder, which are typified by heightened aggression (41). In light of this, and bearing in mind that coadmininstration of COUMATE and DHEAS has previously been shown to increase aggression in mice (21), it was interesting to observe that 39,X^Y^*O mice appeared more overtly aggressive than their 40,XY counterparts.

The neurobiological substrates of the behavioral effects described herein are unknown. *Sts* is expressed predominantly in the thalamus, with lesser expression in the cortex and hindbrain (16). Structural or functional changes in one or more of these brain regions, together with their associated circuitry, may underlie the behavioral effects. We have compared the expression of a number of candidate attentional genes in vehicle and COUMATE-treated mouse frontal cortex and thalamus but have failed to find any significant differences between the two groups to date (Supplement 1).

In rats, lesions of the dorsal anterior cingulate cortex produce specific deficits in accuracy that resemble those seen in COUMATE-treated mice (32), whereas lesions of either the nucleus basalis magnocellularis (42) or the pedunculopontine tegmental nucleus (PPTg) (43) produce a pattern of deficits analogous to that observed in 39,X^Y^*O mice (increased omissions and reduced accuracy at short stimulus durations and reduced premature responding). In rodents, the basal forebrain cholinergic complex (which includes the nucleus basalis magnocellularis) projects directly to the cortex and hippocampus and also to the thalamus, whereas the pontine cholinergic system exerts its influence mainly through intralaminar thalamic nuclei, but it also connects to the basal forebrain and provides a minor innervation of the cortex (44). Hence, our behavioral findings, in combination with previous data indicating links between steroid sulfatase and neurosteroid action on cholinergic systems (45–48), suggest that these manipulations may affect attentional functioning by influencing cholinergic transmission (49,50).

A modulatory role for the steroid sulfatase axis on attention through γ-aminobutyric acid(GABA)-ergic function is also possible: DHEAS is a known negative allosteric modulator of GABA_A_-receptor-gated chloride channels (51), and *Sts* expression correlates with the expression of certain GABA_A_ receptor subunits (52). Infusion of the GABA_A_ receptor agonist muscimol into the subthalamic nucleus in rats (a site of *Sts* expression) produces an array of deficits recapitulating those seen in mice with compromised steroid sulfatase function (i.e., reduced accuracy, slowed correct responses, increased omissions and decreased premature responding) (53). Although any suggestion of a mechanistic link between neurosteroid and acetylcholinergic/GABAergic effects on attention is speculative, it may be pertinent that cholinergic neurons of the basal forebrain exhibit high expression of the GABA_A_ α3 subunit (54), the expression of which is reduced in the attentionally impaired 39,XO mouse (52). It is also noteworthy that 1) α4β2-type nicotinic receptors can control GABAergic transmission in the thalamus (55), 2) the gene encoding the α4 nicotinic receptor subunit is associated with ADHD (56–58), 3) α7-type nicotinic receptors may be found on GABAergic interneurones (59), and 4) α7 nicotinic receptor knockout mice show attention deficits (increased omissions) in the 5-CSRTT (60). In vivo microdialysis in COUMATE-treated and 39,X^Y^*O mice, coupled with pharmacologic manipulations of the acetylcholinergic and GABAergic systems, could be used to test the idea that steroid sulfatase dysfunction causes attentional deficits through perturbations in these systems.

Our data strengthen the case for *STS* as a candidate gene for attentional (dys)function in humans and suggest that it would be worthwhile to investigate association between the gene and ADHD endophenotypes. Recent, as yet unreplicated, work does appear to show an association between *STS* variation and ADHD (61). Our data suggest that variation within *STS* may be particularly associated with the inattentive subtype of ADHD and underlying differences in the structure or function of the thalamocortical circuitry.

## Figures and Tables

**Figure 1:**
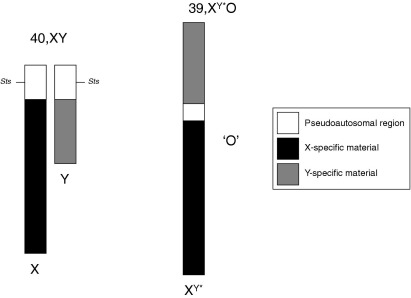
In 39,X^Y^*O mice, an end-to-end fusion of the X and Y chromosome pseudo-autosomal regions results in deletion of both copies of the *Sts* gene.

**Table 1:** Baseline Performance on the 5-Choice Serial Reaction Time Task

Behavioral Parameter	40,XY (*n* = 9)	39,X^Y^*O (*n* = 12)	Effect of Genotype
Number of Trials	74.5 ± 4.1	83.7 ± 3.7	*t*(19) = −1.61, ns
Accuracy (%)	93.3 ± 1.3	95.2 ± .8	*t*(19) = −1.18, ns
Omissions (%)	15.1 ± 1.4	15.8 ± 1.2	*t*(19) = −.38, ns
Correct Response Latency (sec)	.85 ± .06	.91 ± .04	*t*(19) = −.85, ns
Latency to Collect Reinforcer (sec)	3.33 ± .86	2.84 ± .59	*t*(19) = .49, ns
Latency to Consume Reinforcer (sec)	1.98 ± .49	2.00 ± .41	*t*(19) = −.03, ns
Premature Responses	2.1 ± .5	1.3 ± .3	*U* = 54, ns
Nose Pokes Per Trial	1.33 ± .16	1.02 ± .04	*t*(19) = 2.18, *p* = .04
Panel Pushes Per Trial	1.90 ± .14	1.79 ± .17	*t*(19) = .48, ns

**Table 2:** Baseline Performance on the 1-Choice Serial Reaction Time Task

Behavioral Parameter	40,XY (*n* = 9)	39,X^Y^*O (*n* = 12)	Effect of Genotype
Number of Trials	73.3 ± 5.2	77.9 ± 5.2	*t*(19) = −.61, ns
Accuracy (%)	97.8 ± .7	98.3 ± .2	*t*(19) = −.25, ns
Omissions (%)	8.9 ± 1.8	11.1 ± 1.2	*t*(19) = −1.02, ns
Correct Response Latency (sec)	.64 ± .04	.76 ± .05	*t*(19) = −1.74, ns
Latency to Collect Reinforcer (sec)	3.57 ± 1.26	3.36 ± .97	*t*(19) = .13, ns
Latency to Consume Reinforcer (sec)	2.10 ± .63	1.96 ± .43	*t*(19) = .19, ns
Premature Responses	1.4 ± .5	1.7 ± .5	U = 54, ns
Nose Pokes Per Trial	1.62 ± .36	1.10 ± .03	*t*(19) = 1.65, ns
Panel Pushes Per Trial	1.62 ± .12	1.70 ± .16	*t*(19) = −.36, ns
