# Supplementary material for: Converging Pharmacological and Genetic Evidence Indicates a Role for Steroid Sulfatase in Attention
Source: Biol Psychiatry. 2009 Aug 15;66(4):360–7. doi: 10.1016/j.biopsych.2009.01.001 (PMC2720459; doi:10.1016/j.biopsych.2009.01.001)
Supplement: Supplement 1 [file mmc1.pdf]

Supplemental Information

Subjects for genetic study

39,X^{Y*}O mice were produced from two separate crosses: 1) 39,X^{Paf}O (female) x 40,XY* (male) and 2) 39,X^{Paf}O (female) x 39,X^{Y*}O (male). *Paf* is an X-linked mutation involving a small inversion spanning the pseudoautosomal boundary which gives rise to a 'patchy fur' phenotype (1) in heterozygous females (or males), whilst Y* is a Y chromosome that has been hijacked by a non-Y centromere attached distal to the pseudoautosomal region (2, 3). Cross 1 produces three male genotypes: 40,$X^{Paf}X^{Y*}$ (which can be identified through their patchy fur between postnatal days 7-10), 40,X^{Paf}Y* and 39,X^{Y*}O; the latter two genotypes can be differentiated through a polymerase chain reaction (PCR) for the *Sts* gene (see below). Cross 2 produces two male genotypes: 40,$X^{Paf}X^{Y*}$ (which can be identified through their patchy fur between postnatal days 7-10) and 39,X^{Y*}O. 40,XY males were generated from a 40,XY x 40,XX cross. Care was taken to keep the genetic backgrounds for the crosses equivalent (i.e. predominantly MF1 with two C3H strain-derived factors which enable fertility of 39,X^{Y*}O males).

Sts polymerase chain reaction genotyping

Mice were genotyped at weaning and genotyping was confirmed *post mortem*. Tissue was digested in lysis buffer (10mM Tris-HCl pH 8.5, 1.5mM $MgCl_2$, 50mM KCl, 0.45% NP40, 0.45% Tween20, 0.1μg/μl Proteinase K) overnight at 55°C. After denaturation (95°C, 15 mins) and centrifugation (13 000rpm for 5 mins), supernatant was used in the following 25μl PCR reaction: 3.0μl sterile water, 5.0μl 5X buffer, 10.0μl 15mM dNTPS, 5.0μl 5M betaine, 0.25μl forward primer (5' GCTCGCTGACATCATCCTC 3' 300ng/μl stock solution), 0.25μl reverse primer (5' CACCGATGCCCAGGTCGTC 3' 300ng/μl stock solution), 0.5μl Taq polymerase (5U/μl) and 1.0μl DNA solution. PCR conditions were as follows: 94°C for 5 mins, 35 cycles [96°C for 10s, 57°C for 30s, 72°C for 10s], 72°C for 5 mins. The 101bp product was resolved on an ethidium bromide-stained 3% agarose gel.

Animal husbandry

Upon arrival in Cardiff, the mice for the genetic study were treated with Baytril and Septrin antibiotics for one month in a negative-pressure isolator to cure a *Pasteurella pneumotropica* infection prior to being released onto the open racks. Mice for the pharmacological study were group housed (3-5 mice per cage) in a holding room maintained at 21°C ± 2°C and 50% ± 10% humidity, with a 12 hour light-dark cycle (lights

on at 07:00hr). Mice for the genetic study were housed in an identical environment, either singly (due to the tendency of 39,$X^{Y*}O$ mice to fight) or in groups of up to three. Group housed mice for the genetic study were kept with mice of the same genotype. Initially, mice were allowed *ad libitum* access to food and water. Two weeks prior to the onset of behavioural testing mice were placed on a water restriction schedule whilst *ad libitum* access to food was maintained (4 hours access to water for 4 days and 3-4 hours per day thereafter according to the number of mice in the cage). Regular health checks and weighing ensured that mice were not adversely affected by this schedule. Behavioural testing was performed between the hours of 07:00hr and 12:00hr.

Training on the 5-CSRTT

Mice were trained to respond via a nosepoke to a light stimulus presented pseudo-randomly in one of 5 uncovered holes of a standard 9-hole box; stimulus presentation occurred 5s (inter-trial interval, ITI) after initiation of the trial by a ‘panel push’ opposite the response array. A correct response (i.e. a nosepoke in the illuminated hole) resulted in delivery of 20μl of reinforcer behind the panel, the collection of which initiated a second trial. An incorrect response (i.e. a nosepoke in a non-illuminated hole), an omission (i.e. a failure to respond for the duration of the stimulus + 5s) or a premature response (i.e. a response prior to the onset of the stimulus light) resulted in a 5s ‘time-out’ period in which the house-lights were illuminated. This ‘time-out’ could be terminated through a panel push, which initiated a new trial. Mice were given one training session per day, of 20 minutes duration. During training, the stimulus duration was reduced from 32s to a baseline of 1.0s in the following sequence (32s, 16s, 8s, 4s, 2s, 1.8s, 1.6s, 1.4s, 1.2s and 1.0s). The stimulus duration for a given mouse was reduced to the next level once it had achieved stable and high performance at the previous level (i.e. >50 trials, >80% accuracy i.e. ratio of correct:total responses and <30% omissions i.e. no response to <30% of trials initiated).

DHEAS does not influence baseline performance in the 5-CSRTT

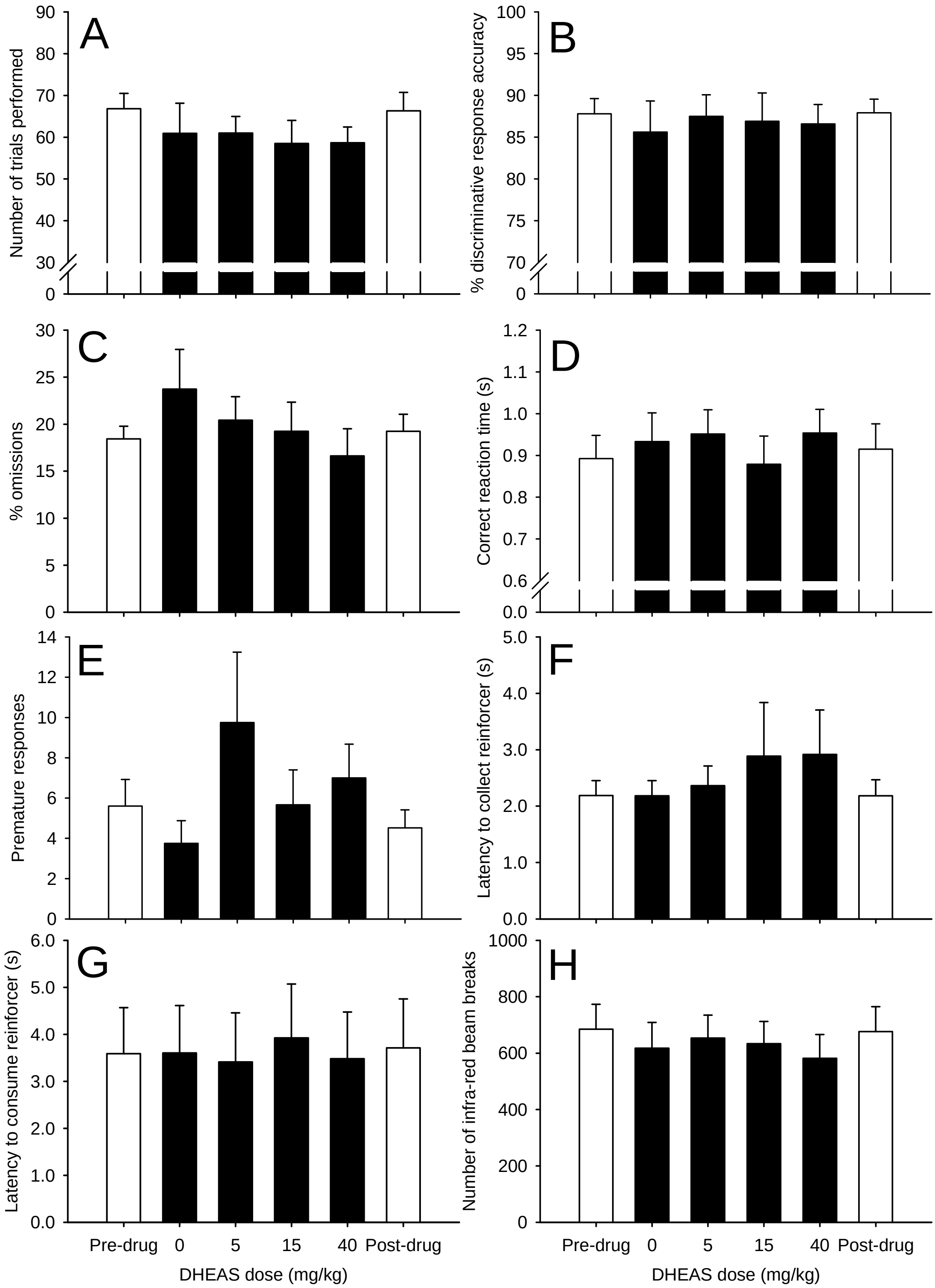

Figure S1. The effect of DHEAS administration on baseline performance in the 5-choice serial reaction time task (5-CSRTT). Data are also shown from baseline sessions prior to drug treatment (pre-drug) and following drug treatment (post-drug). There were no effects of DRUG TREATMENT on: number of trials performed (**A**, F[3,33] = 0.13, n.s.), accuracy (**B**, F[3,33] = 0.04, n.s.), omissions (**C**, F[3,33] = 1.21, n.s.), correct response latency (**D**, F[3,33] = 1.37, n.s.), number of premature responses (**E**, F[3,33] = 1.61, n.s.), latency to collect the reinforcer (**F**, F[3,33] = 0.89, n.s.), latency to consume the reinforcer (**G**, F[3,33] = 1.26, n.s.) or activity (**H**, F[3,33] = 1.18, n.s.).

Gene expression

In order to try and identify a neurobiological correlate for the deficit in accuracy in COUMATE-treated mice relative to their vehicle treated counterparts, gene expression was compared between the groups in previously drug-naïve mice (n = 7 for both groups). We assayed two brain regions where *Sts* expression is highest: the frontal cortex, and the thalamus (4). We examined a number of ADHD candidate genes covering three major neurochemical systems: dopaminergic (*Drd4*, *Drd5*, *Slc6a3*), serotoninergic (*Htr1b*) and cholinergic (*Chrna4*, *Chrna7*) (5). Additionally, the expression of $GABA_A$ receptor subunit genes which are highly expressed in regions of high *Sts* expression, and/or whose expression may be sensitive to steroid sulfatase levels (6) was assessed (*Gabra1*, *Gabra2*, *Gabra3*, *Gabra4*, *Gabra5*, *Gabrd*, *Gabrg2*). Quantitative PCR was performed as described previously (7); primer sequences are available on request. There were no significant differences in expression between vehicle and COUMATE-treated mice for any of the genes assayed (**Figure S2 and Table S1**). The ADHD candidate genes *Dbh* and *Slc6a4* were not expressed in either the frontal cortex or the thalamus, whilst the dopaminergic genes *Drd4*, *Drd5* and *Slc6a3* were expressed at low, or non-existent, levels in the thalamus (C_t values of >30).

Table S1. Statistical comparison of gene expression in the frontal cortex and thalamus regions of vehicle and COUMATE-treated mice (n = 7 for both groups).

Gene of interest	Frontal cortex: Effect of DRUG TREATMENT	Thalamus: Effect of DRUG TREATMENT
Drd4	t[12] = 0.17, n.s.	N/A
Drd5	t[12] = -0.65, n.s.	N/A
Slc6a3	t[12] = -0.12, n.s.	N/A
Htr1b	t[12] = -0.50, n.s.	t[12] = 0.46, n.s.
Chrna4	t[12] = -0.35, n.s.	t[12] = -0.61, n.s.
Chrna7	t[12] = -0.22, n.s.	t[12] = -1.63, n.s.
Snap25	t[12] = -0.36, n.s.	t[12] = -1.50, n.s.
Gabra1	t[12] = -0.41, n.s.	t[12] = -0.37, n.s.
Gabra2	t[12] = 0.40, n.s.	t[12] = -0.75, n.s.
Gabra3	t[12] = -0.01, n.s.	t[12] = -0.18, n.s.
Gabra4	t[12] = -0.34, n.s.	t[12] = -0.34, n.s.
Gabra5	t[12] = 0.04, n.s.	t[12] = 0.38, n.s.
Gabrd	t[12] = 0.20, n.s.	t[12] = -0.33, n.s.
Gabrg2	t[12] = 0.30, n.s.	t[12] = -0.36, n.s.

Figure S2. Expression levels of ADHD candidate genes, and genes putatively sensitive to steroid sulfatase levels in the frontal cortex and thalamus. Data are shown relative to expression levels in vehicle-treated mice.

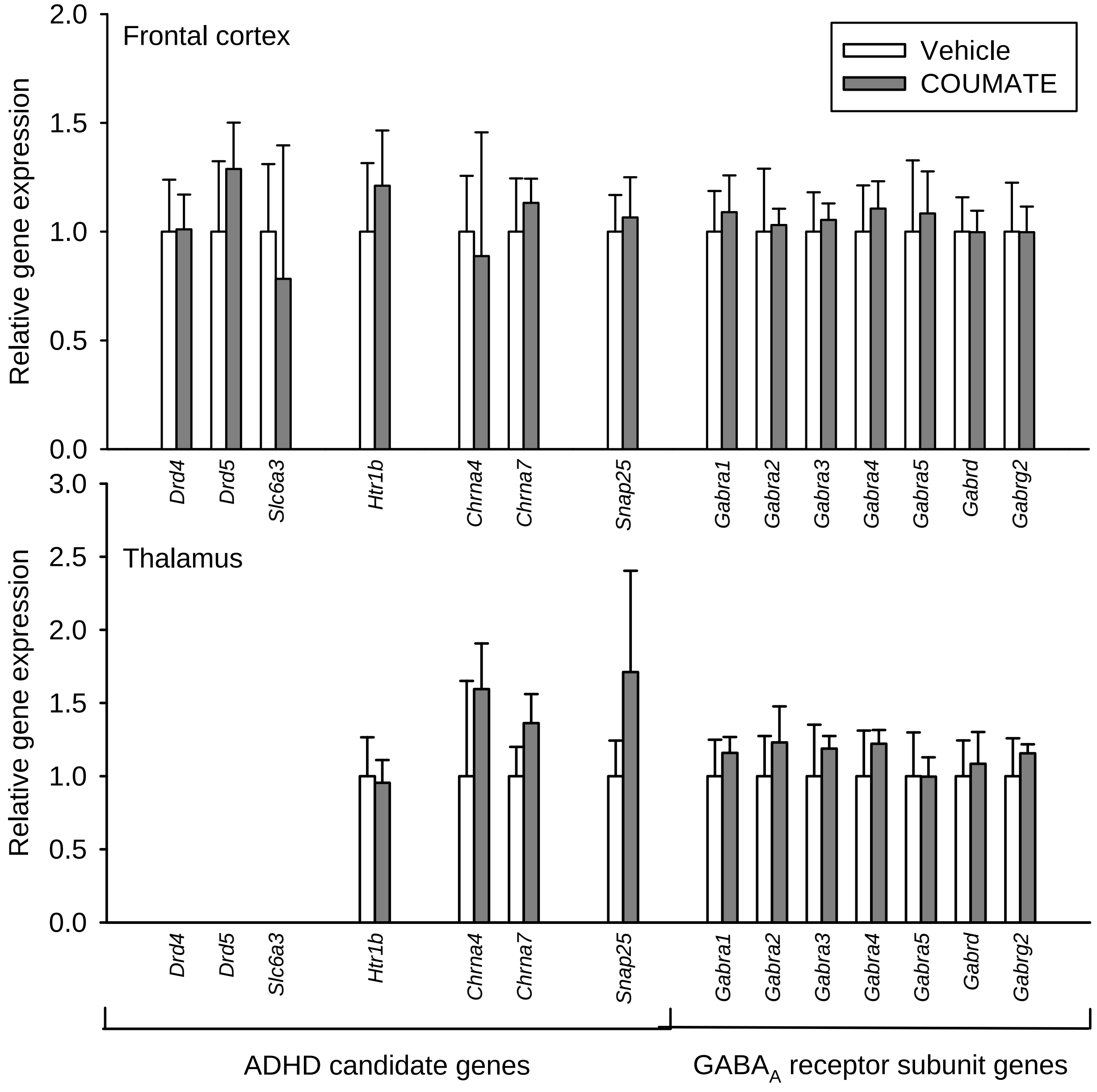

References

1. Korobova O, Lane PW, Perry J, Palmer S, Ashworth A, Davisson MT, Arnheim N (1998): Patchy fur, a mouse coat mutation associated with X-Y nondisjunction, maps to the pseudoautosomal boundary region. *Genomics* 54:556-559.

2. Eicher EM, Hale DW, Hunt PA, Lee BK, Tucker PK, King TR, *et al.* (1991): The mouse Y* chromosome involves a complex rearrangement, including interstitial positioning of the pseudoautosomal region. *Cytogenet Cell Genet* 57:221-230.

3. Burgoyne PS, Mahadevaiah SK, Perry J, Palmer SJ, Ashworth A (1998): The Y* rearrangement in mice: new insights into a perplexing PAR. *Cytogenet Cell Genet* 80:37-40.

4. Compagnone NA, Salido E, Shapiro LJ, Mellon SH (1997): Expression of steroid sulfatase during embryogenesis. *Endocrinology* 138:4768-4773.

5. Faraone SV, Khan SA (2006): Candidate gene studies of attention-deficit/hyperactivity disorder. *J Clin Psychiatry* 67 Suppl 8:13-20.

6. Isles AR, Davies W, Burrmann D, Burgoyne PS, Wilkinson LS (2004): Effects on fear reactivity in XO mice are due to haploinsufficiency of a non-PAR X gene: implications for emotional function in Turner's syndrome. *Hum Mol Genet* 13:1849-1855.

7. Davies W, Humby T, Isles AR, Burgoyne PS, Wilkinson LS (2007): X-monosomy effects on visuospatial attention in mice: a candidate gene and implications for Turner syndrome and attention deficit hyperactivity disorder. *Biol Psychiatry* 61:1351-1360.
